# Evaluation of Epidural Analgesia Use During Labor and Infection in Full-term Neonates Delivered Vaginally

**DOI:** 10.1001/jamanetworkopen.2021.23757

**Published:** 2021-09-15

**Authors:** Lijie Jia, Huimin Cao, Yuna Guo, Ying Shen, Xiaoyu Zhang, Zhou Feng, Jiangruixuan Liu, Zhongcong Xie, Zifeng Xu

**Affiliations:** 1 Department of Anesthesia, the International Peace Maternity and Child Health Hospital, Shanghai Jiao Tong University School of Medicine, Shanghai, China; 2 Department of Obstetrics, the International Peace Maternity and Child Health Hospital, Shanghai Jiao Tong University School of Medicine, Shanghai, China; 3 Department of Nursing, the International Peace Maternity and Child Health Hospital, Shanghai Jiao Tong University School of Medicine, Shanghai, China; 4 Department of Neonatology, the International Peace Maternity and Child Health Hospital, Shanghai Jiao Tong University School of Medicine, Shanghai, China; 5 Department of Anesthesia, Critical Care and Pain Medicine, Massachusetts General Hospital and Harvard Medical School, Boston

## Abstract

**Importance:**

Quantification of potential consequences associated with the use of epidural analgesia during labor could help to improve the safety and quality of labor and delivery care for parturient women.

**Objective:**

To evaluate the association between epidural analgesia use during labor and neonatal infection in a large cohort of parturient women.

**Design, Setting, and Participants:**

This propensity score–matched cohort study was conducted at a university-affiliated hospital in Shanghai, China. Women at full-term pregnancy undergoing vaginal delivery between January 2013 and October 2018 were included in the study. Parturient women who were parous, experiencing premature delivery (gestational age <37 weeks), were pregnant with more than 1 fetus, or had experienced a stillbirth were excluded. Data were analyzed from October 2019 to June 2020.

**Exposures:**

The use of epidural analgesia during labor.

**Main Outcomes and Measures:**

The primary outcome was the incidence of neonatal infection, including neonatal sepsis, neonatal uncharacterized infection, neonatal pneumonia, and neonatal necrotizing enterocolitis reported in the medical record. Secondary outcomes included the incidence of maternal intrapartum fever and histologic chorioamnionitis.

**Results:**

Among 37 786 parturient women included (mean [SD] age, 29.5 [3.0] years), 19 968 (52.8%) received epidural analgesia during labor. In the propensity score–matched cohort (including 15 401 parturient women in each group), use of epidural analgesia was associated with a higher incidence of neonatal infection (absolute risk difference, 2.6%, 95% CI, 2.2%-3.0%; relative risk [RR], 2.43; 95% CI, 2.11-2.78), including higher incidence of sepsis (absolute risk difference, 0.1%, 95% CI, 0.1%-0.2%; RR, 3.50; 95% CI, 1.73-7.07) and uncharacterized infection (absolute risk difference, 2.2%, 95% CI, 1.9% to 2.6%; RR, 2.69; 95% CI, 2.30-3.15), compared with no epidural analgesia use. Use of epidural analgesia was also associated with greater incidence of maternal intrapartum fever (RR, 4.12; 95% CI, 3.78-4.50) and histologic chorioamnionitis (RR, 4.08; 95% CI, 3.59-4.64) compared with no epidural analgesia use.

**Conclusions and Relevance:**

This cohort study found that use of epidural analgesia in full-term nulliparous women undergoing vaginal delivery was associated with an increased risk of neonatal infection, pending further investigation. These findings support efforts to further improve safety and quality of labor and delivery care for parturient women.

## Key Points

**Question:**

Is epidural analgesia during labor associated with neonatal infection?

**Findings:**

In this cohort study of 37 786 parturient women, epidural analgesia was associated with a higher incidence of neonatal infection than no epidural analgesia after propensity score matching.

**Meaning:**

These findings suggest that there is a need for further studies to improve the safety and quality of labor and delivery care in parturient women.

## Teaser

This cohort study uses propensity score matching to examine the association of epidural analgesia use during labor with infection in full-term neonates.

## Introduction

In the US, more than 2 million women each year choose to receive epidural analgesia during labor.^1,2^ The estimated overall prevalence of epidural analgesia use in China is 10%, but in the wealthier economic regions, including Shanghai, the prevalence of epidural analgesia use is 50% to 80%.

Epidural analgesia provides effective relief of labor pain, but the associations between epidural analgesia and maternal and neonatal morbidity are controversial.^1,3,4,5,6,7,8,9^ The risk of maternal fever is 4-fold higher in women receiving epidural analgesia,^10,11,12,13^ but the associations of neonatal outcomes with epidural analgesia–associated maternal fever vary.^14,15,16^ In addition, placental infection, like histologic chorioamnionitis, is associated with epidural analgesia use and may cause intrauterine infection.^17,18^ Whether neonatal outcomes are associated with epidural analgesia remains to be determined, and the association between epidural analgesia use and neonatal infection has yet to be fully elucidated.

Two studies reported no association between epidural analgesia and neonatal events.^19,20^ In contrast, other investigations have reported that epidural analgesia use was associated with increased neonatal sepsis and antibiotic treatment in neonates.^21,22,23^ However, neonatal infection was not the primary outcome of those studies,^21,22,23^ and the number of confirmed sepsis cases was small.^22^ Therefore, the risk of neonatal infection could not be determined owing to limited numbers.^14,15^

To address this, we evaluated the association between epidural analgesia use in labor and neonatal infection in a large cohort of full-term nulliparous women undergoing vaginal delivery. We tested the hypothesis that epidural analgesia is associated with a higher incidence of neonatal infection.

## Methods

### Study Participants

The ethics committee of the International Peace Maternity and Child Health Hospital (IPMCH) in Shanghai, China, approved this study. IPMCH is a prestigious obstetrics and gynecology specialty hospital and performs approximately 15 000 vaginal deliveries per year. Individual consent for this retrospective analysis was waived with permission from the ethics committee because the data were deidentified. The study was reported following the Strengthening the Reporting of Observational Studies in Epidemiology (STROBE) reporting guideline.

Electronic medical record data of deliveries at IPMCH between January 1, 2013, and October 31, 2018, were used for analysis. Only vaginal deliveries were assessed because intrapartum cesarean delivery could be associated with maternal and neonatal morbidity.^24,25^ Parturient women who were parous, experiencing premature delivery (gestational age <37 weeks), or had experienced a stillbirth or multiple gestations were excluded. In addition, records without baseline characteristics (ie, age, weight, and height) or no recorded labor summaries were excluded.

### Exposure and Outcome Definitions

The cost of epidural analgesia (<US $200) and intrapartum care (usually approximately $200-$300) in Shanghai is partly covered by the government (birth plan insurance); therefore, in Shanghai, parturient women may choose not to receive epidural analgesia because of cultural beliefs but not because of socioeconomic status. The women who received epidural analgesia signed informed consent forms. At cervical dilatation of 3 cm or greater and on request, epidural analgesia was initiated for parturient women with no contraindications (eg, coagulation dysfunction). A standard sterile epidural catheter placement was performed at the L3-4 or L2-3 vertebral interspaces with only nurses present, and anesthesiologists wore face masks when placing the catheter. After that, parturient women were assessed hourly, and the infusion rate was adjusted to maintain a T10 sensory level with minimal motor block. Epidural infusions were generally continued through placental expulsion.

Obstetric nurses supervised uncomplicated labor and delivery, and obstetricians were called when assistance was required (eAppendix 1 in the Supplement). After birth, if neonates displayed any unusual symptoms or signs, nurses immediately requested an evaluation from a neonatologist who then selected appropriate laboratory tests and therapies according to neonatological guidelines. Notably, both nurses and neonatologists diagnosed neonatal infection without purposely knowing whether the mothers had epidural analgesia; therefore, ascertainment bias was unlikely.

Data obtained from medical records included maternal age, height, weight (determined within 7 days before delivery), gestational age, parity, group B streptococcus status (positive, negative, or unknown), premature rupture of membranes, medical comorbidities (ie, diabetes, anemia, thyroid dysfunction, and hypertension), labor duration (defined as the length of labor starting from the onset of regular uterine contractions to the delivery of the placenta), intrapartum fever, use of oxytocin, body weight of the newborn, postpartum hemorrhage (defined as ≥500 mL blood loss within 2 hours after delivery), and pathology report among others. Neonatal infections reported in the medical record were collected. Other collected data included Apgar scores (at 1 and 5 minutes after delivery) and neonatal deaths.

The primary outcome was the incidence of neonatal infection occurring within 3 days of birth. Neonatal infection was defined by the occurrence of 1 or more of the following: neonatal sepsis, neonatal uncharacterized infection, neonatal pneumonia, or neonatal necrotizing enterocolitis diagnosed according to the Chinese Classification of Disease and Codes (Version 2.0, *International Statistical Classification of Diseases and Related Health Problems, Tenth Revision* [*ICD-10*]) established by the Chinese government (Table 1). All the diagnostic criteria were required to make the diagnosis. For example, the diagnosis of neonatal sepsis was made if the neonate had positive results in blood or cerebrospinal fluid culture for bacteria, elevated levels of C-reactive protein or procalcitonin or leucocyte count out of reference range, and clinical presentation with temperature outside of reference range, cardiovascular instability, and abnormal skin color. Secondary outcomes were the incidence of maternal and neonatal adverse events, including maternal fever, histologic chorioamnionitis, postpartum hemorrhage, and neonatal Apgar score less than 8 at 1 and 5 minutes after delivery.

**Table 1.** The Diagnostic Criteria of Neonatal Disease According to the Chinese Classification of Disease and Codes

Disease	Code	Diagnostic criteria
Neonatal sepsis	P36.900	Positive results in blood or cerebrospinal fluid culture for bacteria; Raised levels of blood C-reactive protein or procalcitonin, or leucocyte count out of reference range; and Clinical presentation (including abnormal temperature, cardiovascular instability, abnormal skin color).
Neonatal uncharacterized infection	P39.900	Elevated levels of blood C-reactive protein or procalcitonin or leucocyte count out of reference range; Clinical presentation (including temperature outside reference range, cardiovascular instability, abnormal skin color); No confirmed positive results in blood culture; and No confirmed positive results in radiographic examination.
Neonatal pneumonia	P23.900	Respiratory symptoms; Elevated levels of blood C-reactive protein or procalcitonin or leucocyte count out of reference range; and Chest radiographic feature.
Neonatal necrotizing enterocolitis	P77.x01	Feeding intolerance, abdominal distention, or bloody stool and Abdominal radiographic feature.

### Statistical Analysis

Given that there were potential confounding variables in this study, propensity score matching was used to assess the association between epidural analgesia and neonatal infection by constructing a weighted cohort of parturient women with and without epidural analgesia use but similar in demographics and maternal comorbidities before labor and delivery. The propensity score was estimated using a multivariable logistic regression model with epidural analgesia as the dependent variable; covariates included year of delivery, age, body mass index (BMI; calculated as weight in kilograms divided by height in meters squared), premature membrane rupture, comorbidities, and group B streptococcus status. Matching was performed using a 1:1 matching protocol without replacement (greedy-matching algorithm) with a caliper width equal to 0.02 of the SD of the logit of the propensity score.

Before and after propensity score matching, the balance among baseline covariates between the groups was estimated using absolute standardized differences. There was an imbalance between groups assessed by standardized mean difference of more than 10%. In the matched cohort, relative risks (RRs) of primary and secondary outcomes were estimated for women with and without epidural analgesia use. Longer labor duration is recognized as a risk factor of maternal and neonatal morbidity, as described in previous studies.^10,15^ Given that duration of labor was not the baseline characteristics but still a potential contributor to neonatal infection, we performed a binary logistic model that included labor duration to obtain adjusted RRs of primary and secondary outcomes.

Several sensitivity analyses were performed in separate propensity scoring models. Participants were divided based on age (<35 years or ≥35 years), BMI (classified as BMI within reference range [25-29] vs BMI outside reference range [<25 or >29]), and years (2013-2014, 2015-2016, and 2017-2018) (eAppendix 2 in the Supplement). For each subgroup, propensity score matching was used to maintain baseline balance between epidural analgesia use and no epidural analgesia use groups. After propensity score matching, the RRs of primary and secondary outcomes were estimated for parturient women who received epidural analgesia compared with those who did not. The hypothesis tests were 2-sided with significance set at *P* < .05. All statistical analyses were performed using Stata version 15 (Stata Corp) and R statistical software with the statistical package of social sciences version 22.0 (R Project for Statistical Computing). Data were analyzed from October 2019 to June 2020.

## Results

A total of 51 056 parturient women underwent vaginal delivery between 2013 and 2018. Of these, 10 998 were excluded for previous labor and delivery, 1875 were excluded for not reaching term, 352 were excluded for missing demographic characteristics, 30 were excluded for multiple gestations or stillbirth, and 15 were excluded for missing labor record. The final study population included 37 786 parturient women (mean [SD] age, 29.5 [3.0] years). The mean (SD) BMI was 26.1 (2.9), and 1576 women (4.2%) had culture results positive for group B streptococcus. A total of 19 968 women (52.8%) received epidural analgesia. Data before propensity score matching demonstrated that parturient women who received epidural analgesia had a greater incidence of positive results for group B streptococcus (904 women [4.5%] vs 672 women [3.8%]) and greater neonate birth weight (mean [SD], 3.4 [0.4] kg vs 3.3 [0.4] kg) (Table 2). We individually matched 15 401 parturient women who received epidural analgesia to 15 401 parturient women who did not receive epidural analgesia by propensity score (eFigure in the Supplement). Covariates were well-balanced in the propensity-matched cohort (absolute standardized difference, <0.1) (Table 2).

**Table 2.** Parturient Characteristics Before and After Propensity Score Matching

Characteristic	No. (%)			
	Before matching, receipt of epidural analgesia		After matching, receipt of epidural analgesia	
	Yes (n = 19 968)	No (n = 17 818)	Yes (n = 15 401)	No (n = 15 401)
Year^a^				
2013-2014	5863 (29.4)	8028 (45.1)	5733 (37.2)	5726 (37.2)
2015-2016	7460 (37.4)	5305 (29.8)	5213 (33.8)	5228 (33.9)
2017-2018	6645 (33.1)	4485 (25.2)	4455 (28.9)	4447 (28.9)
Age, mean (SD), y	29.6 (3.1)	29.4 (3.1)	29.5 (3.0)	29.5 (3.0)
BMI, mean (SD)	26.1 (3.0)	26.0 (2.9)	26.0 (2.9)	26.0 (2.9)
Comorbidity				
Diabetes	1422 (7.1)	1159 (6.5)	1032 (6.7)	1035 (6.7)
Thyroid dysfunction	508 (2.5)	345 (1.9)	316 (2.1)	316 (2.1)
Hypertension	587 (2.9)	434 (2.4)	412 (2.7)	399 (2.6)
Anemia	734 (3.7)	556 (3.1)	537 (3.5)	529 (3.4)
Premature rupture of membranes	366 (1.8)	319 (1.8)	276 (1.8)	269 (1.8)
Group B streptococcus status^a^				
Positive	904 (4.5)	672 (3.8)	622 (4.0)	631 (4.1)
Negative	16 212 (81.2)	12 923 (72.5)	12 001 (77.9)	11 995 (77.9)
Unknown	2852 (14.3)	4223 (23.7)	2778 (18.0)	2775 (18.0)
Neonate birth weight, mean (SD), kg^a^	3.4 (0.4)	3.3 (0.4)	3.3 (0.3)	3.30 (0.3)

Abbreviation: BMI, body mass index (calculated as weight in kilograms divided by height in meters squared).

^a^ There was an imbalance between groups, as assessed by an absolute standardized mean difference of more than 10%.

In the propensity score–matched cohort, we excluded 26 neonates with congenital diseases (21 with congenital cardiac disease and 5 with persistent pulmonary hypertension) from the analysis of neonatal outcomes, given that there could be an association between congenital disease and neonatal infection. Thus, there were 15 386 neonates in the epidural analgesia group and 15 390 neonates in the no epidural analgesia group included in the final analyses of neonatal outcomes. The epidural analgesia group had a higher incidence of neonatal infection than the no epidural analgesia group (incidence, 674 neonates [4.4%] vs 278 neonates [1.8%]; absolute risk difference, 2.6%; 95% CI, 2.2%-3.0%; RR, 2.43; 95% CI, 2.11-2.78) (Table 3).

**Table 3.** RR of the Primary Outcome in Propensity Score–Matched Neonatal Cohort^a^

Outcome	Event, No. (%)	Absolute risk difference, % (95% CI)	RR (95% CI)	Adjusted RR (95% CI)^b^
Neonatal infection				
Epidural analgesia	674 (4.4)	2.6 (2.2 to 3.0)	2.43 (2.11 to 2.78)	1.81 (1.56 to 2.11)
No epidural analgesia	278 (1.8)	[Reference]	1 [Reference]	1 [Reference]
Sepsis				
Epidural analgesia	35 (0.2)	0.1 (0.1 to 0.2)	3.50 (1.73 to 7.07)	2.51 (1.19 to 5.28)
No epidural analgesia	10 (0.1)	[Reference]	1 [Reference]	1 [Reference]
Uncharacterized infection				
Epidural analgesia	557 (3.6)	2.2 (1.9 to 2.6)	2.69 (2.30 to 3.15)	1.95 (1.64 to 2.31)
No epidural analgesia	207 (1.4)	[Reference]	1 [Reference]	1 [Reference]
Pneumonia				
Epidural analgesia	72 (0.5)	0.1 (−0.0 to 0.3)	1.39 (0.97 to 1.98)	1.19 (0.81 to 1.76)
No epidural analgesia	52 (0.3)	[Reference]	1 [Reference]	1 [Reference]
Necrotizing enterocolitis				
Epidural analgesia	16 (0.1)	0.0 (−0.0 to 0.1)	1.46 (0.68 to 3.13)	1.48 (0.64 to 3.42)
No epidural analgesia	11 (0.1)	[Reference]	1 [Reference]	1 [Reference]

Abbreviation: RR, relative risk.

^a^ Propensity score-matched cohort included 15 386 neonates in the epidural group and 15 390 neonates in the no epidural group, after excluding 26 neonates with congenital diseases (21 neonates with congenital cardiac diseases and 5 neonates with persistent pulmonary hypertension) from the analysis of neonatal outcomes.

^b^ Adjusted RR indicates logistic regression adjustment for labor duration.

The higher incidence of neonatal infection in the epidural analgesia group was associated with a higher incidence of neonatal sepsis (incidence, 35 neonates [0.2%] vs 10 neonates [0.1%]; absolute risk difference, 0.1%; 95% CI, 0.1%-0.2%; RR, 3.50; 95% CI, 1.73-7.07) and higher incidence of neonatal uncharacterized infection (incidence, 557 neonates [3.6%] vs 207 neonates [1.4%]; absolute risk difference, 2.2%; 95% CI, 1.9%-2.6%; RR, 2.69; 95% CI, 2.30-3.15) compared with the no epidural analgesia group (Table 3). Among 45 neonates with sepsis, 27 (60.0%) had gram-positive bacterial infections, 11 (24.4%) had gram-negative bacterial infections, 5 (11.1%) had both gram-positive and gram-negative bacterial infections, 1 (2.2%) had fungal infection, and 1 (2.2%) had viral infection. The main gram-positive pathogen was *Staphylococci* (23 infections [71.9%]), and the main gram-negative pathogen was *Escherichia coli* (15 infections [93.8%]), which were observed in the epidural analgesia and no epidural analgesia groups. Epidural analgesia was not significantly associated with neonatal pneumonia risk or neonatal necrotizing enterocolitis (Table 3). Epidural analgesia was associated with longer duration of labor (eTable 1 in the Supplement). After adjustment for labor duration using logistic regression, epidural analgesia was still associated with neonatal infection (RR, 1.81; 95% CI, 1.56-2.11; Table 3). The mean (SD) hospital stay for neonates with infection in the epidural analgesia group was 7.4 (4.0) days, similar to the mean (SD) hospital stay of 7.2 (3.9) days in the no epidural analgesia group. No neonates died while in the hospital.

Epidural analgesia was associated with higher incidence of maternal temperature greater than 37.5 °C (incidence, 2379 women [15.4%] vs 577 women [3.8%]; RR, 4.12; 95% CI, 3.78-4.50) and increased incidence of histologic chorioamnionitis (incidence, 1139 women [7.4%] vs 274 women [1.8%]; RR, 4.08; 95% CI, 3.59-4.64) compared with the no epidural analgesia group (Table 4). Notably, in this study, only 2546 women (16.5%) in the epidural analgesia group and 1021 women (6.6%) in the no epidural analgesia group underwent histologic evaluation; the rate of histologic chorioamnionitis among placentas evaluated was 1139 placentas (44.7%) in the epidural analgesia group and 274 women (26.8%) of women in the no epidural analgesia group. After adjusting for labor duration, epidural analgesia remained associated with a higher incidence of maternal fever and histological chorioamnionitis (Table 4). Notably, using 38 °C as the cutoff for fever, we found that the association of epidural analgesia and maternal fever remained intact (RR, 7.66; 95% CI, 5.76-10.19). Moreover, in the epidural analgesia group, maternal fever was associated with neonatal infection, including sepsis, uncharacterized infection, and pneumonia, but not necrotizing enterocolitis (eTable 2 in the Supplement).

**Table 4.** RR of Secondary Outcomes in the Propensity Score–Matched Cohort

Outcome	Events, No. (%)	RR (95% CI)	Adjusted RR (95% CI)^a^
**Maternal outcome** ^b^			
Fever			
Epidural analgesia	2379 (15.4)	4.12 (3.78-4.50)	3.37 (3.05-3.72)
No epidural analgesia	577 (3.8)	1 [Reference]	1 [Reference]
Chorioamnionitis			
Epidural analgesia	1139 (7.4)	4.16 (3.65-4.73)	2.98 (2.59-3.43)
No epidural analgesia	274 (1.8)	1 [Reference]	1 [Reference]
Postpartum hemorrhage			
Epidural analgesia	226 (1.5)	0.95 (0.79-1.14)	0.79 (0.64-0.96)
No epidural analgesia	238 (1.6)	1 [Reference]	1 [Reference]
**Neonatal outcome** ^**c**^			
Apgar score <8 at 1 min			
Epidural analgesia	159 (1.0)	1.42 (1.12-1.81)	1.06 (0.82-1.39)
No epidural analgesia	112 (0.7)	1 [Reference]	1 [Reference]
Apgar score <8 at 5 min			
Epidural analgesia	19 (0.1)	1.27 (0.64-2.49)	0.94 (0.45-1.96)
No epidural analgesia	15 (0.1)	1 [Reference]	1 [Reference]

Abbreviation: RR, relative risk.

^a^ Adjusted RR, logistic regression adjustment for labor duration.

^b^ Propensity score–matched cohort included 15 401 parturient women in the epidural group and 15 401 parturient women in the non-epidural group.

^c^ Twenty-six neonates with congenital diseases were excluded from analyses of neonatal outcomes analysis, and 15 386 neonates in the epidural group and 15 390 neonates in the no epidural group were included.

The incidence of postpartum hemorrhage did not significantly differ between the two groups (incidence, 226 women [1.5%] vs 238 women [1.6%]) (Table 4). Epidural analgesia was also associated with lower Apgar scores at 1 minute after delivery compared with the no epidural analgesia group (incidence of Apgar score <8, 159 neonates [1.0%] vs 112 neonates [0.7%]; RR, 1.42; 95% CI, 1.12-1.81), although this was not significant after adjusting for labor duration (adjusted RR, 1.06; 95% CI, 0.82-1.39). There was no significant difference between the groups in Apgar scores at 5 minutes after delivery (incidence of Apgar score <8, 19 neonates [0.1%] vs 15 neonates [0.1%]) (Table 4). Moreover, the epidural analgesia group had greater interventions, including use of oxytocin (incidence, 10 533 women [68.4%] vs 8535 women [55.4%]) and instrument delivery (incidence, 1899 women [12.3%] vs 1514 women [9.8%]).

### Sensitivity Analyses

Before sensitivity analyses, parturient women in the epidural analgesia group were matched with parturient women in the no epidural analgesia group for each divided group using propensity score weight. Covariates were well balanced in the propensity score–matched cohort, without statistically significant differences. In sensitivity analyses, epidural analgesia was associated with a higher risk of neonatal infection in parturient women stratified by age, BMI, and year (Figure). Epidural analgesia was also associated with a higher risk of maternal intrapartum fever and histologic chorioamnionitis in each stratified group (eTables 3-5 in the Supplement). The RRs among the 352 women missing BMI data are presented in eTable 6 in the Supplement.

**Figure.**
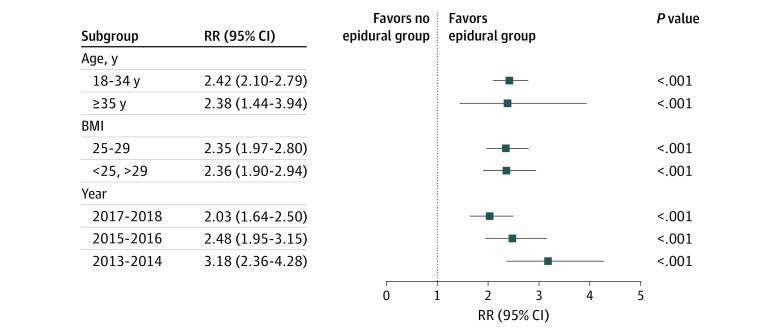
Sensitivity Analyses of the Associations of Neonatal Infection With the Use of Epidural Analgesia According to Maternal Age, Body Mass Index (BMI), and Year BMI is calculated as weight in kilograms divided by height in meters squared. Squares indicate point estimates for relative risks compared with the no epidural analgesia group; horizontal lines, 95% CIs. Separate propensity score matching was used for each subgroup.

## Discussion

In this propensity score–matched cohort study, parturient women who received epidural analgesia during labor experienced an increased risk of neonatal infection compared with women who did not receive epidural analgesia, which was associated with a higher incidence of neonatal sepsis and neonatal uncharacterized infection. Sensitivity analyses yielded consistent results across age, BMI, and date. Longer labor duration is associated with increased risk of both maternal and neonatal infections,^1,26^ and we observed an association of longer labor duration with use of epidural analgesia. However, after adjustment for labor duration, epidural analgesia remained associated with a higher risk of neonatal infection.

Neonatal sepsis was diagnosed based on positive results in blood or cerebrospinal fluid culture for bacteria, elevated levels of C-reactive protein or procalcitonin, leucocyte count out of reference range, and clinical presentation (eg, temperature outside of reference range, cardiovascular instability). Previous studies have observed that epidural analgesia is associated with increased odds of evaluation for neonatal sepsis and treatment with antibiotics.^22,23,27,28,29,30,31,32,33^ Still, epidural analgesia was not associated with increased risk of confirmed sepsis,^22,23,27,28,29,30,31,32,33^ possibly owing to the small numbers of neonatal sepsis cases. The number of confirmed sepsis cases was only 3 neonates (0.3%) in the epidural analgesia group and 1 neonate (0.2%) in the no epidural analgesia group in a study by Lieberman et al, 1 neonate (0.1%) in the epidural analgesia group and no neonates in the no epidural analgesia group reported in a study by Goetzl et al,^30^ and no neonates in either group in several other studies.^18,27,28^ The smaller sizes of these studies meant fewer total cases of neonatal infection, potentially preventing the detection of an association between epidural analgesia and increased risk of neonatal infection. Consistent with this, a systematic review and meta-analysis by Jansen et al^14^ determined that the low incidence of sepsis cases may explain the lack of an association between epidural analgesia and neonatal bacteremia in prior studies. In our study, 674 neonates in the epidural analgesia group and 278 neonates in the no epidural analgesia group developed a neonatal infection. Moreover, approximately 50% of the total cohort underwent epidural analgesia in our study, so the balance between groups was conducive to detecting differences.

Nurses and neonatologists evaluated neonates without purposely knowing whether the mother had received epidural analgesia because there have been no studies suggesting the potential association between epidural analgesia and neonatal infection. Therefore, it was less likely that ascertainment bias accounted for the observed association between epidural analgesia and neonatal infection in the present study.

Sepsis developing within 72 hours of life typically results from vertical transmission from mother to infant.^34^ Given the difficulty of obtaining blood or cerebrospinal fluid for bacteria culture, the neonatal uncharacterized infection in this study, diagnosed according to clinical presentations and laboratory tests (ie, C-reactive protein, procalcitonin, and white blood cell levels) without identified causes, could be a stage of infectious or inflammatory response rather than a definitive infection, attributable to either bacteriuric intraamniotic infection or sterile intraamniotic inflammation.^17,35,36^ Nevertheless, the causes of neonatal infection remain elusive. Future studies should focus on the determination of the underlying mechanism by which neonatal infection occurs.

Maternal fever and chorioamnionitis are associated with fetal inflammatory response and neonatal sepsis.^14,15^ However, many studies suggest that placental inflammation is not an indicator of infection.^37^ However, placental inflammation may indicate and may cause epidural analgesia–associated maternal fever. We analyzed maternal fever and histologic chorioamnionitis as secondary outcomes in this present study. The definition of maternal intrapartum fever was not unified in previous studies.^38,39^ The relatively strict threshold used in this study was the threshold used in our institution, and our data were consistent with earlier reports that the increased risks of maternal intrapartum fever associated with epidural analgesia were between 2.9- and 14.5-fold higher.^16^ Our results suggest that maternal fever could contribute, at least partially, to the association between epidural analgesia and neonatal infection. In addition, we and others^18,40^ used leukocyte infiltration of the chorionic plate as the marker of histologic chorioamnionitis^17^; however, without placental culture findings, we could not determine whether the histologic chorioamnionitis observed in our study was infectious in origin or sterile inflammation.

### Limitations

This study has several limitations. First, histological examination of the placenta was performed when parturient women presented clinical symptoms of chorioamnionitis. In this study, only 16.5% of women in the epidural analgesia group and 6.6% of women in the no epidural analgesia group underwent histologic evaluation; and the rate of histologic chorioamnionitis among placentas evaluated were 44.7% of women in the epidural analgesia group and 26.8% of women in the no epidural analgesia group. Therefore, we did not investigate the association of histologic chorioamnionitis with neonatal infection. However, this limitation did not alter the study’s conclusion because the nurses and neonatologists evaluate neonates based on the observation of the neonates but not based on the results of pathologic examination of the mother’s placentas (usually reported 1 week after specimen collection). Second, we did not include parturient women who underwent intrapartum cesarean delivery in this study because intrapartum cesarean delivery is associated with neonatal infection.^24,25^ However, the findings from this study are still clinically useful when obstetricians counsel a parturient woman regarding epidural analgesia because most parturient women have scheduled vaginal delivery rather than cesarean delivery. Third, we could not obtain the exact numbers of vaginal examinations in the participants, which is associated with neonatal infection.^41^ However, the numbers of vaginal examinations were associated with labor duration because it was performed every 2 hours during the labor according to the hospital policy, and longer labor duration was associated with epidural analgesia in our study. Moreover, although the use of oxytocin and instrument delivery was more common in the epidural analgesia group than the no epidural analgesia group, the exact associations of vaginal examination, use of oxytocin, and instrument delivery with neonatal infection were not investigated in this present study. We will perform factorial designed case-control observations to assess the role of these factors associated with epidural analgesia in neonatal infection in the future. Additionally, this study only included nulliparous women. Future research will extend to multiparous women.

## Conclusions

This cohort study found that receipt of epidural analgesia in labor was associated with an increased risk of neonatal infection among term nulliparous women undergoing vaginal delivery. We also found that the use of epidural analgesia was associated with a higher risk of maternal intrapartum fever and histologic chorioamnionitis. Although this study found an association of epidural analgesia use and higher incidence of neonatal infection, the overall incidence of neonatal infection was low, even in the parturient women receiving epidural analgesia. Moreover, neonatal infection could be associated with maternal fever, chorioamnionitis, more vaginal examinations, and the use of oxytocin and instrument delivery. Additionally, the observed neonatal infections were not associated with increased morbidity and mortality, consistent with the previous studies that have found that epidural analgesia is not associated with worse Apgar scores or neonatal intensive care unit admissions.^42^ Thus, the findings from this study should not be the reason for refusing epidural analgesia but should lead to more studies to improve the quality and safety of labor and delivery care, including epidural analgesia, in parturient women.

## Notes

Supplement. **eAppendix 1.** Exposure During Labor and Delivery **eAppendix 2.** Sensitivity Analysis **eTable 1.** Labor Duration in Epidural Analgesia Group and No Epidural Analgesia Group **eTable 2.** Relative Risk of Neonatal Infection in Epidural Analgesia Group With and Without Maternal Fever **eTable 3.** Relative Risk of Secondary Outcomes in the Propensity Score–Matched Age Subgroup **eTable 4.** Relative Risk of Secondary Outcomes in the Propensity Score–Matched BMI Subgroup **eTable 5.** Relative Risk of Secondary Outcomes in the Propensity Score–Matched Time Subgroup **eTable 6.** Relative Risk of Primary Outcome in 352 Women Missing BMI Data **eFigure.** The Distribution of Propensity Scores Before and After Propensity Scoring Match
eReferences
