# Supplementary material for: Evaluation of Epidural Analgesia Use During Labor and Infection in Full-term Neonates Delivered Vaginally
Source: JAMA Netw Open. 2021 Sep 15;4(9):e2123757. doi: 10.1001/jamanetworkopen.2021.23757 (PMC8444029; doi:10.1001/jamanetworkopen.2021.23757)
Supplement: Supplement. — eAppendix 1. Exposure During Labor and Delivery eAppendix 2. Sensitivity Analysis eTable 1. Labor Duration in Epidural Analgesia Group and No Epidural Analgesia Group eTable 2. Relative Risk of Neonatal Infection in Epidural Analgesia Group With and Without Maternal Fever eTable 3. Relative Risk of Secondary Outcomes in the Propensity Score–Matched Age Subgroup eTable 4. Relative Risk of Secondary Outcomes in the Propensity Score–Matched BMI Subgroup eTable 5. Relative Risk of Secondary Outcomes in the Propensity Score–Matched Time Subgroup eTable 6. Relative Risk of Primary Outcome in 352 Women Missing BMI Data eFigure. The Distribution of Propensity Scores Before and After Propensity Scoring Match eReferences [file jamanetwopen-e2123757-s001.pdf]

Supplemental Online Content

Jia L, Cao H, Guo Y, et al. Evaluation of epidural analgesia use during labor and infection in full-term neonates delivered vaginally. *JAMA Netw Open*. 2021;4(9):e2123757. doi:10.1001/jamanetworkopen.2021.23757

eAppendix 1. Exposure During Labor and Delivery
eAppendix 2. Sensitivity Analysis
eTable 1. Labor Duration in Epidural Analgesia Group and No Epidural Analgesia Group
eTable 2. Relative Risk of Neonatal Infection in Epidural Analgesia Group With and Without Maternal Fever
eTable 3. Relative Risk of Secondary Outcomes in the Propensity Score–Matched Age Subgroup
eTable 4. Relative Risk of Secondary Outcomes in the Propensity Score–Matched BMI Subgroup
eTable 5. Relative Risk of Secondary Outcomes in the Propensity Score–Matched Time Subgroup
eTable 6. Relative Risk of Primary Outcome in 352 Women Missing BMI Data
eFigure. The Distribution of Propensity Scores Before and After Propensity Scoring Match
eReferences

This supplemental material has been provided by the authors to give readers additional information about their work.

© 2021 Jia L et al. *JAMA Network Open.*

eAppendix 1. Exposure During Labor and Delivery

Obstetric nurses supervised normal labor and delivery, and obstetricians were called when assistance was required. Temperatures were recorded when parturients arrived at the labor unit and were assessed every two hours by nursing staff during labor and delivery. Body temperature larger than 37.5°C was recorded. Given that the temperature elevation (e.g., > 37.5°C) was associated with adverse outcomes in parturients,[1] our hospital used 37.5°C as a definition for fever, consistent with other studies.[1,2] Pethidine (100 mg) was intramuscularly administrated at the first request for pain relief before 3 cm cervical dilatation. Parturient received ampicillin plus gentamicin (or cephalosporins plus gentamicin if parturients were allergic to ampicillin) if their temperature ≥ 38.0°C with one or more of the following presentations: maternal heart rate ≥100 bpm, fetal heart rate ≥160 bpm, and white blood count ≥15*10^9/L with neutrophil percentage >90%. The parturient also received the antibiotics if their rupture-of-membrane duration was longer than 24 hours or tested positive for group B streptococcus. Histological examination of the placenta was performed when parturients presented clinical symptoms of chorioamnionitis. The pathological evidence of chorioamnionitis, amnionitis, chorionic vasculitis, and funisitis were examined.

© 2021 Jia L et al. *JAMA Network Open.*

eAppendix 2. Sensitivity Analysis

Participants were divided based on age (<35 years or ≥35 years), body mass index [BMI, calculated as weight in kilograms divided by height in meters squared; normal BMI: 25–29 or abnormal BMI: <25 and >29], and date (2013-2014, 2015-2016, and 2017-2018). Either greater age or greater BMI was associated with higher incidence of neonatal events and the obstetric, anesthetic and neonatal care might improve over time.[3-5] In the present study, the number of high BMI (>29) was 4,468 and the number of low BMI (<25) was 12,025, which is not comparable. But the number of normal BMI (25-29) was 14,309 and the number of abnormal BMI was16,493, which is comparable.

© 2021 Jia L et al. *JAMA Network Open.*

eTable 1. Labor Duration in Epidural Analgesia Group and No Epidural Analgesia Group

Labor duration	Epidural	No-Epidural	*P*-value
Mean ± Standard deviations (minute)	515 ± 201	352 ± 162	/
Median (interquartile ranges) (minute)	490 (365-635)	310 (235-423)	<0.001
> 600 minutes (n, %)	4513 (29.3)	1293 (8.4)	<0.001

The labor duration was not normally distributed in either epidural group or no-epidural group. The Median and numbers > 600 minutes were compared using the nonparametric test and chi-square test, respectively.

© 2021 Jia L et al. *JAMA Network Open.*

eTable 2. Relative Risk of Neonatal Infection in Epidural Analgesia Group With and Without Maternal Fever†

Outcome	Neonates with event, n (%)	RR (95% CI)	Adjusted RR (95% CI)
Neonatal infection			
Maternal fever	378 (15.9)	7.01 (6.06-8.11)	7.35 (6.25-8.64)
No maternal fever	296 (2.3)	Reference	Reference
Sepsis			
Maternal fever	26 (1.1)	15.85 (7.44-33.78)	15.01 (6.93-32.49)
No maternal fever	9 (0.1)	Reference	Reference
Uncharacterized infection			
Maternal fever	330 (13.9)	7.98 (6.78-9.39)	8.11 (6.79-9.70)
No maternal fever	227 (1.7)	Reference	Reference
Pneumonia			
Maternal fever	21 (0.9)	2.26 (1.36-3.79)	2.17 (1.29-3.66)
No maternal fever	51 (0.4)	Reference	Reference
Necrotizing Enterocolitis			
Maternal fever	3 (0.1)	1.27 (0.36-4.44)	1.40 (0.39-5.04)
No maternal fever	13 (0.1)	Reference	Reference

† The epidural cohort included 2,372 neonates with maternal intrapartum fever and 13,014 neonates without maternal intrapartum fever. RR denotes relative risk. Adjusted RR indicates logistic regression adjustment for labor duration.

© 2021 Jia L et al. *JAMA Network Open.*

eTable 3. Relative Risk of Secondary Outcomes in the Propensity Score–Matched Age Subgroup

Characteristic	Age ≥35 years		Age <35 years	
	No. of Participants with event	Relative risk (95% CI)	No. of Participants with event	Relative risk (95% CI)
Maternal outcome				
Fever				
Epidural	167 (18.1)	5.06 (3.52-7.27)	2,195 (15.2)	4.07 (3.71-4.45)
No-Epidural	33 (3.6)	Reference	540 (3.7)	Reference
Chorioamnionitis				
Epidural	97 (10.5)	5.11 (3.15-8.28)	1,085 (7.5)	4.16 (3.64-4.75)
No-Epidural	19 (2.1)	Reference	261 (1.8)	Reference
Postpartum hemorrhage				
Epidural	18 (2.0)	0.82 (0.44-1.52)	202 (1.4)	0.92 (0.76-1.11)
No-Epidural	22 (2.4)	Reference	220 (1.5)	Reference
Neonatal outcome†				
Apgar score at 1 min < 8				
Epidural	19 (2.1)	1.73 (0.83-3.61)	158 (1.1)	1.51 (1.18-1.92)
No-Epidural	11 (1.2)	Reference	105 (0.7)	Reference
Apgar score at 5 min < 8				
Epidural	4 (0.4)	4.00 (0.45-35.76)	23 (0.2)	1.53 (0.80-2.94)
No-Epidural	1 (0.1)	Reference	15 (0.1)	Reference

† Twenty-eight neonates with congenital diseases (21 with congenital heart disease, 6 with persistent pulmonary hypertension, and 1 with hereditary metabolic disease) in the age ≥35 years subgroup and 1 with congenital heart disease in the age <35 years subgroup were excluded from outcomes analysis.

© 2021 Jia L et al. *JAMA Network Open.*

eTable 4. Relative Risk of Secondary Outcomes in the Propensity Score–Matched BMI Subgroup

Characteristic	BMI <25 or >29		BMI 25-29	
	No. of Participants with event	Relative risk (95% CI)	No. of Participants with event	Relative risk (95% CI)
Maternal outcome				
Fever				
Epidural	945 (14.3)	3.89 (3.39-4.46)	1,411 (16.2)	4.34 (3.86-4.88)
No-Epidural	243 (3.7)	Reference	325 (3.7)	Reference
Chorioamnionitis				
Epidural	498 (7.5)	3.46 (2.88-4.15)	666 (7.7)	4.72 (3.95-5.65)
No-Epidural	144 (2.2)	Reference	141 (1.6)	Reference
Postpartum hemorrhage				
Epidural	100 (1.5)	1.10 (0.83-1.46)	137 (1.8)	0.90 (0.72-1.13)
No-Epidural	91 (1.4)	Reference	152 (1.7)	Reference
Neonatal outcome†				
Apgar score at 1 min < 8				
Epidural	73 (1.1)	1.46 (1.02-2.09)	98 (1.1)	1.61 (1.17-2.21)
No-Epidural	50 (0.8)	Reference	61 (0.7)	Reference
Apgar score at 5 min < 8				
Epidural	12 (0.2)	1.33 (0.56-3.16)	12 (0.1)	1.50 (0.61-3.67)
No-Epidural	9 (0.1)	Reference	8 (0.1)	Reference

† Thirteen neonates with congenital diseases (9 with congenital heart disease, 3 with persistent pulmonary hypertension, and 1 with hereditary metabolic disease) in the BMI <25 or >29 subgroup and 15 with congenital diseases (14 with congenital heart disease and 1 with persistent pulmonary hypertension) in the BMI 25-29 subgroup were excluded from outcomes analysis. BMI, body mass index is calculated as weight in kilograms divided by height in meters squared.

© 2021 Jia L et al. *JAMA Network Open.*

eTable 5. Relative Risk of Secondary Outcomes in the Propensity Score–Matched Time Subgroup

Characteristic	2013-2014		2015-2016		2017-2018	
	No. of participants with Event	Relative risk (95% CI)	No. of participants with event	Relative risk (95% CI)	No. of participants with event	Relative risk (95% CI)
Maternal outcome						
Fever						
Epidural	554 (9.7)	3.85 (3.21-4.60)	789 (15.2)	4.05 (3.48-4.71)	970 (22.3)	4.20 (3.66-4.82)
No-Epidural	144 (2.5)	Reference	195 (3.8)	Reference	231(5.3)	Reference
Chorioamnionitis						
Epidural	275 (4.8)	2.72 (2.17-3.41)	392 (7.6)	5.03 (3.95-6.39)	465 (10.7)	4.47 (3.63-5.51)
No-Epidural	101 (1.8)	Reference	78 (1.5)	Reference	104 (2.4)	Reference
Postpartum Hemorrhage						
Epidural	79 (1.4)	0.95 (0.70-1.29)	67 (1.3)	0.91 (0.65-1.26)	78 (1.8)	0.95 (0.70-1.29)
No-Epidural	83 (1.5)	Reference	74 (1.4)	Reference	82 (1.9)	Reference
Neonatal Outcome†						
Apgar score at 1 Min < 8						
Epidural	78 (1.4)	2.23 (1.50-3.31)	3 (0.8)	0.87 (0.57-1.33)	50 (1.2)	1.56 (1.01-2.43)
No-Epidural	35 (0.6)	Reference	45 (0.9)	Reference	32 (0.7)	Reference
Apgar score at 5 min < 8						
Epidural	13 (0.2)	2.60 (0.93-7.29)	5 (0.1)	0.63 (0.21-1.91)	3 (0.1)	0.75 (0.17-3.35)
No-Epidural	5 (0.1)	Reference	8 (0.2)	Reference	4 (0.1)	Reference

† Four neonates with congenital diseases (2 with congenital heart disease and 2 with persistent pulmonary hypertension) in the 2013-2014 subgroup, 7 with congenital diseases (6 with congenital heart disease and 1 with hereditary metabolic disease) in the 2015-2016 subgroup and 16 with congenital diseases (14 with congenital heart disease and 2 with persistent pulmonary hypertension) in the 2017-2018 subgroup were excluded from outcomes analysis.

© 2021 Jia L et al. *JAMA Network Open.*

eTable 6. Relative Risk of Primary Outcome in 352 Women Missing BMI Data

Outcome	Neonates with event, n (%)	RR (95% CI)	Adjusted RR† (95% CI)
Neonatal infection			
Epidural	8 (4.7)	2.17 (0.66-7.06)	2.12 (0.59-8.09)
No-Epidural	4 (2.2)	Reference	Reference

† Adjusted RR indicates logistic regression adjustment for labor duration. BMI, body mass index is calculated as weight in kilograms divided by height in meters squared.

© 2021 Jia L et al. *JAMA Network Open.*

eFigure. The Distribution of Propensity Scores Before and After Propensity Scoring Match

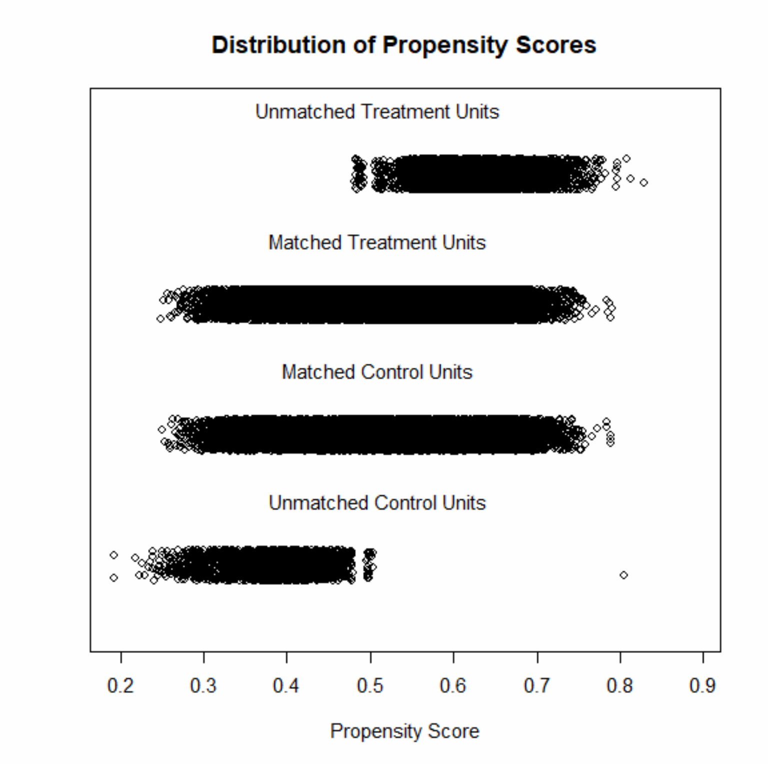

The Treatment Units were in the epidural group, and the Control Units were in the no-epidural group.

© 2021 Jia L et al. *JAMA Network Open.*

eReferences

1. Greenwell EA, Wyshak G, Ringer SA, Johnson LC, Rivkin MJ, Lieberman E. Intrapartum temperature elevation, epidural use, and adverse outcome in term infants. *Pediatrics.* 2012;129(2):e447-454.
2. Yancey MK, Zhang J, Schwarz J, Dietrich CS, 3rd, Klebanoff M. Labor epidural analgesia and intrapartum maternal hyperthermia. *Obstet Gynecol.* 2001;98(5 Pt 1):763-770.
3. Kaul B, Vallejo M, Ramanathan S, Mandell G. Epidural labor analgesia and neonatal sepsis evaluation rate: a quality improvement study. *Anesth Analg.* 2001;93(4):986-990.
4. Jacobsson B, Ladfors L, Milsom I. Advanced maternal age and adverse perinatal outcome. *Obstet Gynecol.* 2004;104(4):727-733.
5. Heslehurst N, Simpson H, Ells LJ, et al. The impact of maternal BMI status on pregnancy outcomes with immediate short-term obstetric resource implications: a meta-analysis. *Obes Rev.* 2008;9(6):635-683.

© 2021 Jia L et al. *JAMA Network Open.*
